# Small RNA-based prediction of hybrid performance in maize

**DOI:** 10.1186/s12864-018-4708-8

**Published:** 2018-05-21

**Authors:** Felix Seifert, Alexander Thiemann, Tobias A. Schrag, Dominika Rybka, Albrecht E. Melchinger, Matthias Frisch, Stefan Scholten

**Affiliations:** 1 Developmental Biology, Biocenter Klein Flottbek, University of Hamburg, 22609 Hamburg, Germany; 2 Institute for Plant Breeding, Seed Science and Population Genetics, Quantitative Genetics and Genomics of Crops, University of Hohenheim, Fruwirthstrasse 21, 70599 Stuttgart, Germany; 3 Institute of Agronomy and Plant Breeding II, Justus Liebig University, 35392 Giessen, Germany

**Keywords:** Hybrid trait prediction, Small RNA, Hybrid performance, Grain yield, Maize, Epigenetics, Transcriptome, SNP

## Abstract

**Background:**

Small RNA (sRNA) sequences are known to have a broad impact on gene regulation by various mechanisms. Their performance for the prediction of hybrid traits has not yet been analyzed. Our objective was to analyze the relation of parental sRNA expression with the performance of their hybrids, to develop a sRNA-based prediction approach, and to compare it to more common SNP and mRNA transcript based predictions using a factorial mating scheme of a maize hybrid breeding program.

**Results:**

Correlation of genomic differences and messenger RNA (mRNA) or sRNA expression differences between parental lines with hybrid performance of their hybrids revealed that sRNAs showed an inverse relationship in contrast to the other two data types. We associated differences for SNPs, mRNA and sRNA expression between parental inbred lines with the performance of their hybrid combinations and developed two prediction approaches using distance measures based on associated markers. Cross-validations revealed parental differences in sRNA expression to be strong predictors for hybrid performance for grain yield in maize, comparable to genomic and mRNA data. The integration of both positively and negatively associated markers in the prediction approaches enhanced the prediction accurary. The associated sRNAs belong predominantly to the canonical size classes of 22- and 24-nt that show specific genomic mapping characteristics.

**Conclusion:**

Expression profiles of sRNA are a promising alternative to SNPs or mRNA expression profiles for hybrid prediction, especially for plant species without reference genome or transcriptome information. The characteristics of the sRNAs we identified suggest that association studies based on breeding populations facilitate the identification of sRNAs involved in hybrid performance.

**Electronic supplementary material:**

The online version of this article (10.1186/s12864-018-4708-8) contains supplementary material, which is available to authorized users.

## Background

A key objective of modern crop breeding is to generate hybrids to increase yield by exploiting heterosis, as well as take advantage of a uniform F1 population. The generation of large numbers of inbred lines does not constitute a bottleneck through the application of doubled-haploid technology [1], but it is economically not feasible to phenotype the hybrids resulting from all possible inbred line combinations. In addition, neither the parental per se performance nor the genetic distance between the parental genomes are perfect predictors for the selection of optimal inbred line combinations [2]. Thus, the selection process needs to be supported by prediction approaches based on genomic markers (e.g. AFLP, RFLP, SSR or SNP) [3, 4], transcriptome profiles [5–10], metabolomic [9–11] or phenomic [12] markers as predictors, that are assessed in the parental inbred lines.

Epigenetic variations have been suggested to be important components for complex traits such as crop yield [13]. Genome-wide epigenetic states, such as DNA methylation or chromatin modifications, affect phenotypes including complex traits, such as yield, without any changes to the genome sequence [14]. It has been shown that hybrids of *Arabidopsis* ecotypes and rice subspecies showed substantial epigenetic variations at the level of DNA methylation, histone modifications and small RNAs (sRNAs) [15, 16]. *Arabidopsis* hybrids of near-isogenic but epigenetically diverse parents exhibit substantial heterosis for various traits [17]. Artificial selection from an isogenic plant population over multiple generations resulted in plants with superior phenotypic performance, which could be stably inherited over generations [18]. These results suggest that epigenetics has the potential to enhance future plant breeding as well as to provide useful markers [18, 19]. Non-coding sRNAs have been shown to be key regulators of epigenetic states [19] and sRNA expression levels undergo drastic changes after hybridization [20–22]. The mechanisms of trans-chromosomal methylation and demethylation have been associated with small RNA expression level differences between parental inbred lines [23]. These relations suggest that parental sRNAs play a major role in setting genome-wide changes in the epigenetic landscape through hybridization. In turn, parental sRNAs are likely to reflect these changes to a certain extend and thus we assume that they might be promising markers for the prediction of hybrid traits.

Our objective was to investigate the predictiction of hybrid performance (HP) for grain yield (GY) using parental sRNA expression profiles. We used next-generation sRNA sequencing data of whole 7-day-old seedlings of 21 elite maize inbred lines from which 98 (7 × 14) hybrid crosses were generated. A previous study revealed high prediction accuracy with distance measures based on trait-associated mRNAs [5]. Here, we developed this association approach further by integrating the identification of negatively trait-associated markers in addition to positively associated markers. We introduce a distance measure, which combines the positively and negatively associated markers in one measure and two prediction approaches, based on simple or multivariate linear regression (MLR). We used the new association approach to identify SNPs, mRNAs or sRNAs associated with HP for GY and compared prediction accuracies of the various marker types by cross-validation. Further, we characterized the sRNA population that showed an association with HP for GY concerning size distribution, genomic location and relation to genomic features.

## Methods

### Plant material and phenotyping

The plant material for this association study represents a half-diallel mating scheme of of 21 maize inbred lines (7 Flint and 14 Dent) from the breeding program of the University of Hohenheim, Germany, with 98 hybrids resulting from the factorial mating scheme of Dent by Flint lines [24]. The set of Flint lines is composed by four lines with European Flint background (F037, F039, F043, F047) and three with Flint/Lancaster background (L028, L035, L043). The Dent lines include eight lines with an Iowa Stiff Stalk Synthetic (S028, S036, S044, S046, S049, S050, S058, S067) and six with an Iodent background (P033, P040, P046, P048, P063, P066). Field trials were conducted to collect the phenotypic data at five locations for the inbred lines in 2003 and 2004 and at six locations for the hybrids in 2002 [5, 25]. Field data for GY were measured in Mg ha^− 1^ with adjustment to 155 g kg^− 1^ grain moisture (Additional file 1: Table S1).

For transcriptome expression analysis and sRNA sequencing all 21 inbred lines were grown under controlled conditions (25 °C, 16 h day, 8 h night, 70% air humidity) for seven days, the whole seedlings including roots were flash-frozen in liquid nitrogen. Five biological individuals of the same genotype were pooled before RNA isolation.

### SNP data

SNP data were generated with the Illumina MaizeSNP50 chip [26] in the study of Frascaroli et al. [4].

### Microarray transcriptome expression data generation and analysis

Microarray gene expression data was generated on the 46 k maize oligo nucleotide array [27]. RNA-probe synthesis and microarray analysis are described in the study of Thiemann et al. [28]. All expression data has been deposited in the NCBI GEO under accession number GSE17754.

### Small RNA isolation, sequencing and sequencing data processing

Small RNA isolation, sequencing experiments as well as sequencing data processing and normalization are described in the study of Seifert et al. [22]. All sequence data has been deposited in the NCBI GEO under accession number GSE51662. Details on the sRNA sequencing data are given in Additional file 2: Table S2.

### Identification of discriminative markers

Polymorphic SNPs, where nucleotides of at least one line differed from the remaining lines, were considered as discriminative markers. For mRNA, differential expression is defined as described for microarray analysis for at least one inbred line combination. For sRNA, differential expression between inbred lines is defined as a minimum expression of the lower expressed parent of 0.5 rpmqn and a two-fold expression change towards the higher expressed parent. In case the expression of the lower parent is below 0.5 rpmqn, the higher parent needs to have at least an expression of 1 rpmqn to consider a sRNA as differentially expressed.

### Correlation of genomic and mRNA/sRNA expression differences with hybrid performance

The euclidean distances D_e_ (1) were calculated for all three data types (SNP, mRNA, sRNA) as the sum of marker differences for all markers that are differential in at least one inbred line combination. The differences for the data types are calculated for the combination of the inbred lines i and j, with d_s_(i,j) being the expression difference for a specific sRNA, mRNA, or SNP.

1 $$ {D}_e\left(i,j\right)=\sqrt{\sum \limits_{s=1}^n{d}_s\left(i,j\right)} $$

The expression difference d_s_ for sRNA and mRNA expression data c_s_ between the lines i and j is calculated as follows:

2 $$ {d}_s\left(i,j\right)={\left({c}_s(i)-{c}_s(j)\right)}^2 $$

The difference d_s_ for SNP data with c_s_ being the actual sequence between the lines i and j is calculated as follows:

3 $$ {d}_s\left(i,j\right)=\left\{\begin{array}{l}0\kern0.5em \mathrm{if}\kern0.5em {c}_s(i)\kern0.5em \ne \kern0.5em {c}_s(j)\\ {}1\kern0.5em \mathrm{if}\kern0.5em {c}_s(i)\kern0.5em =\kern0.5em {c}_s(j)\end{array}\right. $$

### Marker trait association

Associations of markers with the traits HP for GY were established analogous to Frisch et al. [5] by separating the hybrids into classes of low and high trait values (L, H) with equal size and binomial testing. For each individual marker (SNP, mRNA, sRNA) the number of hybrids with differential marker (sequence, expression) between the inbred parents was counted for both classes L and H as o_L_ and o_H_ respectively. With the null hypothesis that differential expression occurs with the same probability for both classes, the probability P_s_ (4) of a marker being associated to the trait was estimated via the binomial distribution probability function. This function depends on the number of hybrids whose inbred lines exhibit differential expression for the given sRNA in the classes L and H:

4 $$ {P}_s=\sum \limits_{k={k}_{min}}^n{Bin}_{n,p}(k)\kern1em with\kern1em n=\left({o}_H+{o}_L\right),p= 0.5 $$

with

5 $$ n=\left({o}_H+{o}_L\right) $$

and setting equal probability for association with L and H by *p* = 0.5. The parameter k_min_ depending on positive (6) or negative (7) association:

6 $$ {k}_{min}={o}_L\kern1em if\kern1em {o}_L>{o}_H $$

7 $$ {k}_{min}={o}_H\kern1em if\kern1em {o}_L<={o}_H $$

All markers with *p*-values lower than the probability threshold after adjustment for multiple testing via FDR correction at 0.05 [29] were considered as associated to the specific trait (HP for GY). The certainty of the association against random artifacts was tested by permutation analyses (100 runs) of the datasets by randomly re-assigned hybrid trait values to the hybrids.

### Calculation of distances for trait associated markers

We calculated trait-associated marker binary distances for two inbred lines i and j and a defined set of n markers as follows:

8 $$ {D}_b\left(i,j\right)=\sqrt{\frac{1}{n}\sum \limits_{s= 1}^n{x}_s} $$

with x_s_ being set to 1 for differential markers between the two inbred lines and 0 otherwise.

To integrate the opposing binary distances for positively and negatively associated markers in one distance measure, we developed the combined binary distance which integrates the binary distance for n_pos_ positively associated markers D_b,pos_ and n_neg_ binary distance for negatively associated markers D_b,neg_ for the two inbred lines i and j as follows:

9 $$ {D}_{b, com}\left(i,j\right)=\frac{D_{b, pos}\left(i,j\right)\cdot {n}_{pos}+\left( 1\hbox{-} {D}_{b, neg}\left(i,j\right)\right)\cdot {n}_{neg}}{n_{pos}+{n}_{neg}} $$

### Prediction of hybrid performance

The prediction of HP was performed after Frisch et al. [5] using a linear regression model. In contrast to Frisch et al. [5] both positively and negatively associated markers were integrated by using the combined binary distance (Formula 9) as follows:

10 $$ Y\left(i,j\right)={\beta}_0+{\beta}_0\ast {D}_b\left(i,j\right) $$

Additionally a HP prediction based using multivariate linear regression (MLR) was performed as follows:

11 $$ {Y}_m\left(i,j\right)={\beta}_0+{\beta}_1\cdot {D}_{b, pos}\left(i,j\right)+{\beta}_2\cdot {D}_{b, neg}\left(i,j\right)+{\beta}_3\cdot mf\left(i,j\right)+{\beta}_4\cdot {m}_d\left(i,j\right) $$

Four predictors were included in the MLR, including the binary distances of positively associated markers D_b,pos_, as well as negatively associated markers D_b,neg_. The third predictor m_f_ represents the fraction of differential positively associated markers n_pos_(i,j) of all differential associated markers, given by the sum of n_pos_(i,j) and differential negatively associated markers n_neg_(i,j), between the two lines the two lines i and j:

12 $$ mf\left(i,j\right)=\frac{n_{pos}\left(i,j\right)}{n_{pos}\left(i,j\right)+{n}_{neg}\left(i,j\right)} $$

The fourth predictor m_d_ represents the dominance of positively or negatively differential associated markers between the lines i and j, given as n_pos_(i,j) and n_neg_(i,j), to the difference of the positively and negatively associated markers (n_pos_, n_neg_) defined as follows:

13 $$ {m}_d\left(i,j\right)=\frac{n_{pos}\left(i,j\right)-{n}_{neg}\left(i,j\right)}{n_{pos}-{n}_{neg}+0.1} $$

The denominator is incremented by 0.1 to avoid division by zero, if the sets of positively and negatively associated markers are equally large. This predictor includes information about the number of differential associated markers in relation to all associated markers.

We performed three different prediction scenarios as described by Schrag et al. [3]. In the scenario of type-0 prediction none of the two parents were used in test-crosses, whereas for type-1 prediction one of the two parents has been used, for type-2 prediction test-cross data for both parental inbred lines is available. For all prediction types (type-2, type-1, type-0) 3 Flint and 5 Dent lines were chosen randomly to form the estimation set. In the type-1 prediction one of the two heterotic groups was randomly selected to define the lines with known test-cross data. All lines not selected in the estimation set were used as validation set to assess the prediction accuracy as the Pearson correlation coefficient of observed and predicted values for HP for GY. The prediction accuracy was determined in 100 cross-validation runs.

### sRNA differential expression analysis between heterotic groups

We used DESeq2 [30] to call differentially expressed sRNAs in support of the threshold-based differential sRNA expression results from 7 Flint × 14 Dents inbred lines without biological replication. We explored the differential expression between the Dent and Flint heterotic groups by setting three genetically most related lines within the groups, according to the genomic grouping of Frisch et al. [5], as replicates and analyzed two different sets (Set1: Flint: F039, F043, F047; Dent: S036, S050, S058 / Set2: Flint: L024, L035, L043; Dent: P033, P040, P066). The sRNAs of each set were filtered for sequences with a summed read count from all replicates of 10 or higher. The differential expression was tested using DESeq2 [30] individually for each set with lines assorted by the heterotic groups. All sRNAs with FDR < 5% were considered as differentially expressed.

We analyzed the differentially expressed sRNAs for known miRNA sequences as well as overlap with hybrid performance associated-sRNAs (hpa-sRNAs) that we identified by marker-trait association analysis. The fractions of differentially expressed hpa-sRNAs of the two sets were compared to the fractions of threshold-based differential hpa-sRNAs from the 9 corresponding inbred line combinations.

### sRNA enrichment analyses

The *p*-values for enrichment and depletion of HP for GY associated sRNAs of specific sequence length were computed by bootstrap analysis as described in Seifert et al. [22].

### Reference genome mapping of sRNAs and annotation analysis

The mapping of sRNAs to the B73 reference genome (AGPv4; July 2017) [31] as well as annotation analysis were perfomed as described in Seifert et al. [22].

## Results

### Correlation of genomic, mRNA and sRNA expression differences with hybrid performance

**Fig. 1:**
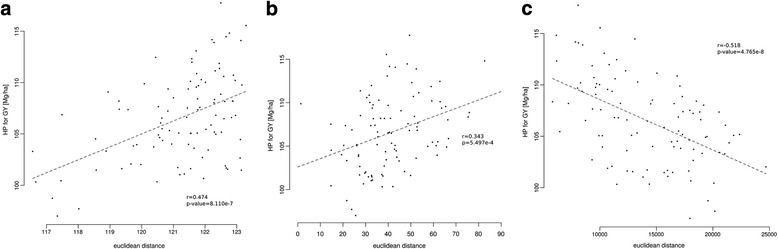
Correlation r of hybrid performance for grain yield with parental differences for SNP (**a**), mRNA (**b**) or sRNA (**c**) data

For all three data types (SNP, mRNA, sRNA) we correlated the sum of differences between inbred line combinations with HP for GY to test for relations of parental differences and HP for GY. All individual features with differences between at least one inbred line combination of Flint and Dent lines were included in the analysis. In total, 32,330 (55.9%) SNPs, 12,414 (28.6%) of the mRNAs and 178,753 (0.6%) of the sRNAs were included in the analysis. The correlation of SNP differences and mRNA differences between inbred line combinations with HP for GY in their hybrids resulted in moderate (*r* = 0.474, *p* = 8.110 *×* 10^− 7^, Fig. 1a) and weak (*r* = 0.343, *p* = 5.5 × 10^− 4^, Fig. 1b) positive correlations, respectively. The correlation of sRNA expression differences between inbred parents and HP for GY in their hybrids results in a moderate negative correlation (*r* = − 0.518, *p* = 4.675 × 10^− 8^, Fig. 1c) opposing to the correlations for SNP and mRNA, showing that differing information are covered by sRNA expression profiles.

### Association of differential data types with hybrid performance

**Fig. 2:**
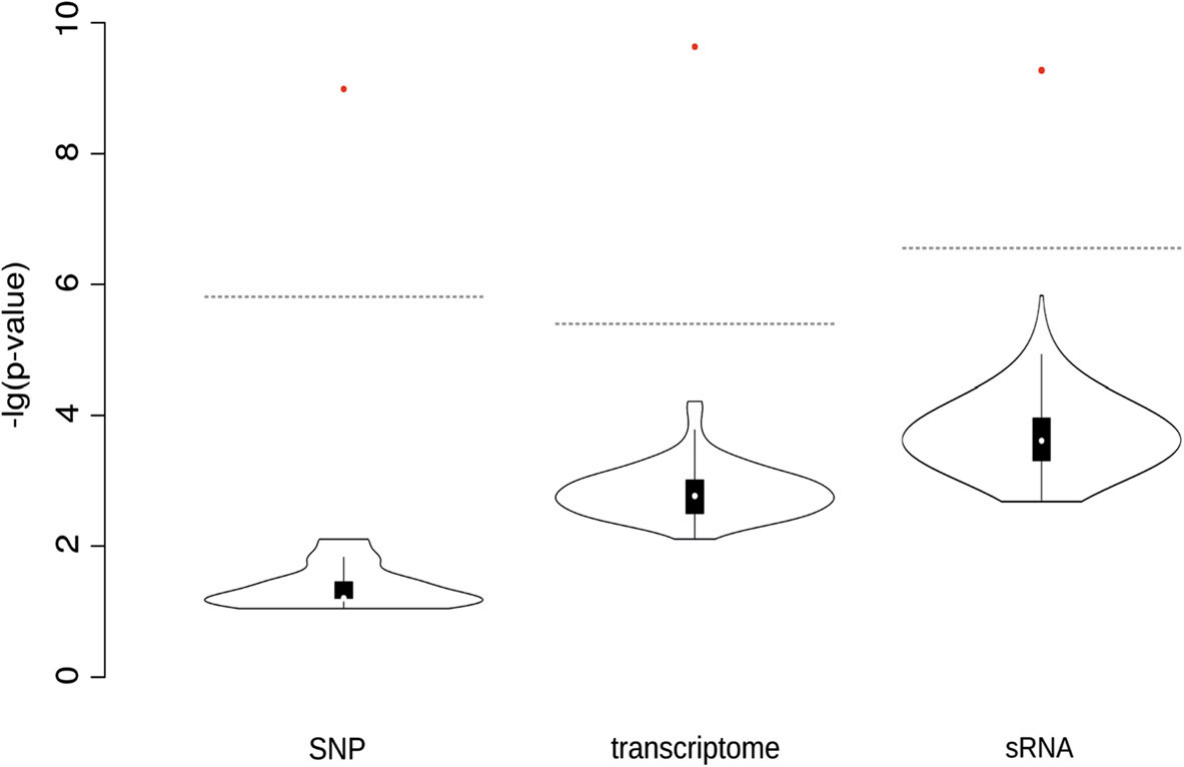
Permutation analysis with shuffled hybrid trait values with SNP, transcriptome (mRNA), or sRNA data. The lowest *p*-values of each permutation run (black violin plot) and of the actual genotype-trait allocation (red dot) are represented. The dotted line indicates the threshold to reach significance at 5% FDR

To account for the different directions of overall correlation between parental variation of the various data types and hybrid performance, we analyzed the number and direction of associated variation. Consistent to the correlation analysis, all individual features of the data types were included in the analysis showing differences between at least one pair of Flint and Dent inbred lines. The association of SNPs with HP for GY results in 5191 associated SNPs, representing 16.1% of the candidate SNPs. These associated SNPs are almost equally distributed into positively (54.3%) and negatively (45.7%) associated SNPs. There were 729 mRNAs significantly associated with HP for GY, representing 5.9% of all candidate mRNAs. In contrast to the associated SNPs, there is a clear major fraction of 82.17% transcripts with positive association with HP for GY. The distribution of 7142 sRNAs associated with HP for GY, representing a subset of 4.0% of the candidate sRNAs, showed a larger fraction of positively associated sRNAs (61.9%). We named the sRNAs, which showed a significant association with HP for GY based on the full set of inbred lines (5% FDR, binomial test), hybrid performance associated-sRNAs (hpa-sRNAs). The numbers as well as fractions of positively and negatively associated markers for all three data types (SNP, mRNA, sRNA) are listed in Table 1. To control against random associations by chance alone, we performed permutation tests, shuffling the hybrid trait values. These tests resulted in the loss of all associations - all 100 permutations by far did not reach the significance level required to call any associated marker for all three data types (Fig. 2).

**Table 1:** Number of positively/negatively associated markers (SNP, mRNA, sRNA) with HP for GY. Fraction of positively or negatively associated markers of all markers are given in brackets

Data type	Associated markers	pos. associated markers	neg. associated markers
SNP	4941	2687 (54.381%)	2254 (45.618%)
mRNA	729	599 (82.167%)	130 (17.833%)
sRNA	7142	4423 (61.929%)	2719 (38.071%)

### Correlation of marker distances with hybrid performance

We performed separate correlations for all predictors with HP for GY. The five predictors are the binary distances for positively associated markers (D_b,pos_), negatively associated markers (D_b,neg_), the combined binary distance (D_b,com_) and the two additional predictors m_f_ and m_d_, which represent additional information about the size of D_b,pos_ and D_b,neg_. In addition, the correlation strength of the MLR was tested. For both SNPs and sRNAs the binary distance based on D_b,neg_ result in stronger correlations (*r* = − 0.801 and *r* = − 0.831) than on D_b,pos_. The opposite result was obtained for mRNA data, where the correlation for D_b,pos_ was stronger (*r* = 0.819) than for D_b,neg_ (− 0.794). The predictor m_f_ results in equally strong correlations as for the positively associated markers for SNP and sRNA markers but stronger correlations for mRNA markers (*r* = 0.846). In comparison m_d_ does not results in superior correlations to the stronger binary distance D_b,neg_ for SNPs and sRNAs or D_b,pos_ for mRNAs. Whereas the combined binary distance (D_b,com_) performs for SNPs and mRNAs equally good as the best correlation of the other four predictors, the correlation for the MLR outperforms all five predictors with a slight increase for SNPs (*r* = 0.804) but distinct increases for both mRNAs (*r* = 0.861) and sRNAs (*r* = 0.857). The results show that the negatively associated markers as binary distance (D_b,neg_) are highly related to HP for GY and the combination of information on positively and negatively associated markers in D_b,com_ or the MLR for all three data types always outperform correlations based on D_b,pos_ alone. All correlation results are listed in Table 2.

**Table 2:** Correlation of marker-based distances for all associated markers with HP for GY

	Correlation coefficient (r)					
Data type	Binary distance for pos. Associated (D_b,pos_)	Binary distance for neg. Associated (D_b,neg_)	Fraction of pos. to all associated markers (m_f_)	Dominance of pos. vs. neg. Associated markers (m_d_)	Combined binary distance (D_b,com_)	Multivariate linear regression (predictors: D_b,pos_, D_b,neg_, m_f_, m_d_)
SNP	0.787	− 0.801	0.788	0.793	0.797	0.804
mRNA	0.819	−0.794	0.846	0.801	0.84	0.861
sRNA	0.796	−0.831	0.800	0.811	0.818	0.857

### Prediction of hybrid performance for grain yield

**Fig. 3:**
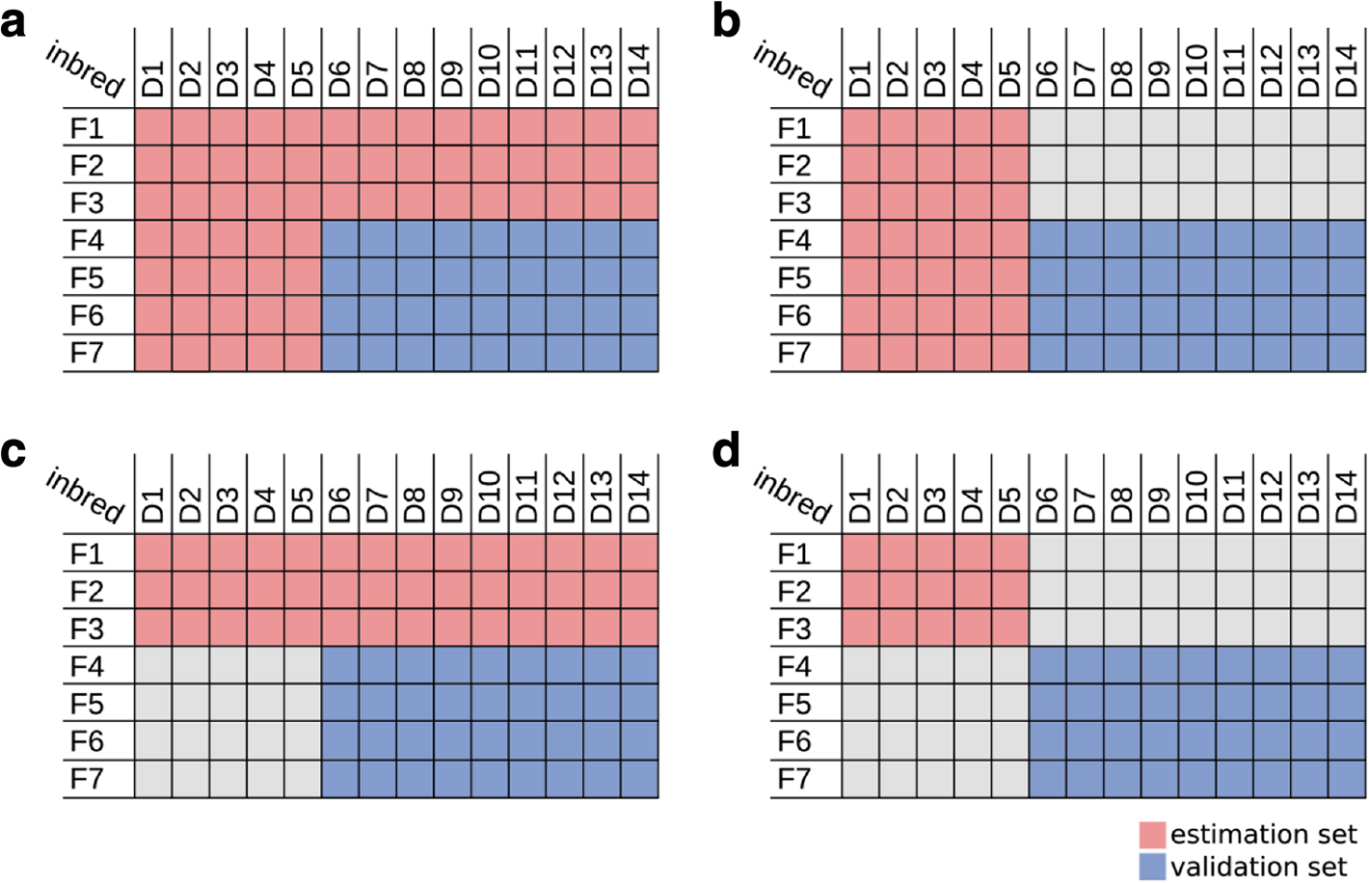
Prediction types (**a**) type-2 prediction, both parents have test crosses; (**b, c**) type-1 prediction, only one of the parental groups has test crosses; (**d**) type-0 prediction, none of the parents has been tested

We evaluated marker-based predictions in 100 cross-validation runs by randomly selecting a subset of 5 Dent and 3 Flint inbred lines as estimation sets. Three prediction schemes were distinguished to evaluate the prediction accuracy of sRNA vs. SNP or mRNA marker-based prediction of HP. For type-2 prediction both parents, type-1 one parent and type-0 none of the parents have been evaluated in test-crosses. The three prediction types are schematically shown in Fig. 3. We preformed predictions based on binary distances of positively (D_b,pos_) or negatively (D_b,neg_) associated markers, the combined binary distance (D_b,com_) as well as a MLR-based prediction.

Overall the type-2 prediction performs best for all three data types. The drop of prediction accuracy is mild from type-2 to type-1 prediction, but drastically from type-1 to type-0 prediction for all three data types (Additional file 3: Figure S1A-C).

For all three data types the MLR-based prediction resulted in the highest mean prediction accuracies for all three data types for type-2 prediction, as well as for type-1 prediction using mRNAs as markers. For type-1 prediction using SNP and sRNA as markers, as well as for type-0 prediction for SNP and sRNAs the predictions using the combined binary distance (D_b,com_) revealed the highest mean prediction accuracies. The type-0 prediction for mRNAs performed best using the binary distance of positively associated mRNAs (D_b,pos_, Table 3).

**Table 3:** Mean Prediction accuracies and standard deviation for marker-based predictions (SNP, mRNA, sRNA) of HP for GY. In case not all 100 cross-validation runs resulted with associated markers in the estimation set and thus a prediction was not possible, the numbers of successful predictions is given in brackets

		Prediction accuracy			
Marker type	Prediction type	pos. Associated marker distance (D_b,pos_)	neg. Associated marker distances (D_b,neg_)	Combined marker distance (D_b,com_)	Multivariate linear regression
SNP	type-2	0.767 ± 0.111	0.798 ± 0.068	0.773 ± 0.103	0.799 ± 0.079
SNP	type-1	0.720 ± 0.113	0.675 ± 0.254	0.726 ± 0.124	0.681 ± 0.198
SNP	type−0	0.53 ± 0.241 (80)	0.455 ± 0.397 (33)	0.562 ± 0.253 (78)	0.044 ± 0.334 (39)
mRNA	type-2	0.712 ± 0.073	0.634 ± 0.119	0.787 ± 0.065	0.824 ± 0.054
mRNA	type-1	0.670 ± 0.086	0.534 ± 0.177	0.720 ± 0.077	0.735 ± 0.108
mRNA	type−0	0.498 ± 0.174	0.115 ± 0.295 (98)	0.460 ± 0.207	0.325 ± 0.263
sRNA	type-2	0.82 ± 0.082	0.803 ± 0.099	0.837 ± 0.074	0.843 ± 0.084
sRNA	type-1	0.731 ± 0.109	0.697 ± 0.172	0.753 ± 0.100	0.712 ± 0.189
sRNA	type−0	0.368 ± 0.331	0.269 ± 0.321	0.440 ± 0.302	0.116 ± 0.396

**Fig. 4:**
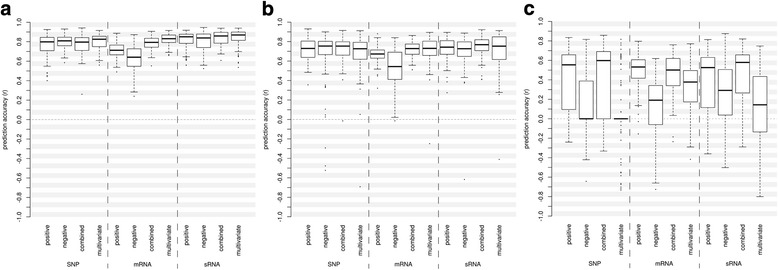
Prediction accuracy for SNP, mRNA and sRNA based prediction of hybrid performance for grain yield for (**a**) type-2 prediction, (**b**) type-1 prediction, (**c**) type-0 prediction

For type-2 predictions, with test-cross information from both parents, the MLR-based prediction using sRNAs as markers outperformed all the other prediction methods (Fig. 4a, Table 3). The standard deviation of the MLR-based predictions, using mRNAs as markers, resulted in a smaller standard deviation than for sRNAs (Table 3). The boxplots generated from the 100 cross-validation runs show that only a few outliers were generating a bias in the standard deviation (Fig. 4a). With test-crosses from only one parent (type-1) the combined binary distance (D_b,com_) using sRNAs as markers performed best (Fig. 4b, Table 3). The prediction without tested parents was strongest using the positively associated mRNAs (D_b,pos_) and showed the least variation. The type-0 predictions based on positively associated (D_b,pos_) SNPs or the combined binary distance (D_b,com_) generated from positively and negatively associated SNPs revealed higher mean prediction accuracies, but had a remarkably higher variation and did not result in predictions in all cross-validation runs (Fig. 4c, Table 3).

### Differential expression of sRNAs between heterotic groups

Our prediction models used distance measures, which integrate parental expression variation to a distinct value for the respective hybrids. In our factorial mating scheme the parents of each hybrid always belong to a different heterotic group. To support the differential sRNA expression between the parental lines we used DESeq2 [30, 32] as an alternative statistical approach and set three genetically most related lines of each heterotic group as replicates. Two sets of inbred lines were analyzed by DESeq2. Overall, the number of DESeq2-based differential expressed known microRNAs (miRNAs) was very low. In one set 5 miRNAs (zma-miR164b-3p, zma-miR156k-5p, zma-miR2118b, zma-miR397a-5p, zma-miR397b-5p) were called differential, whereby the latter two have identical sequences. The second set revealed no differentially expressed miRNAs. The number of hpa-sRNAs called by DESeq2 and the number of differentially expressed hpa-sRNAs between the respective lines based on our initial threshold-based approach are shown in Table 4 for the two sets of inbred lines. Although the absolute numbers of differentially expressed hpa-sRNAs vary, the relations of positively and negatively hpa-sRNAs between the two sets of inbred lines coincided for both approaches.

**Table 4:** Differential expression of hpa-sRNAs between the heterotic groups. Range and mean numbers of threshold-based differential hpa-sRNAs from the 9 inbred line combinations are given

	DESeq2 # DE hpa-RNAs		Threshold-based # DE hpa-sRNAs	
Set# and inbred lines	positively	negatively	positively	negatively
Set1 Flint: F039, F043, F047 Dent: S036, S050, S058	20	672	35–220 mean 140.67	868–1928 mean 1375.56
Set2: - Flint: L024, L035, L043 - Dent: P033, P040, P066	829	0	1956–2284 mean 2086.56	25–217 mean 119.78

### Genomic characterization of sRNAs associated with HP for GY

**Fig. 5:**
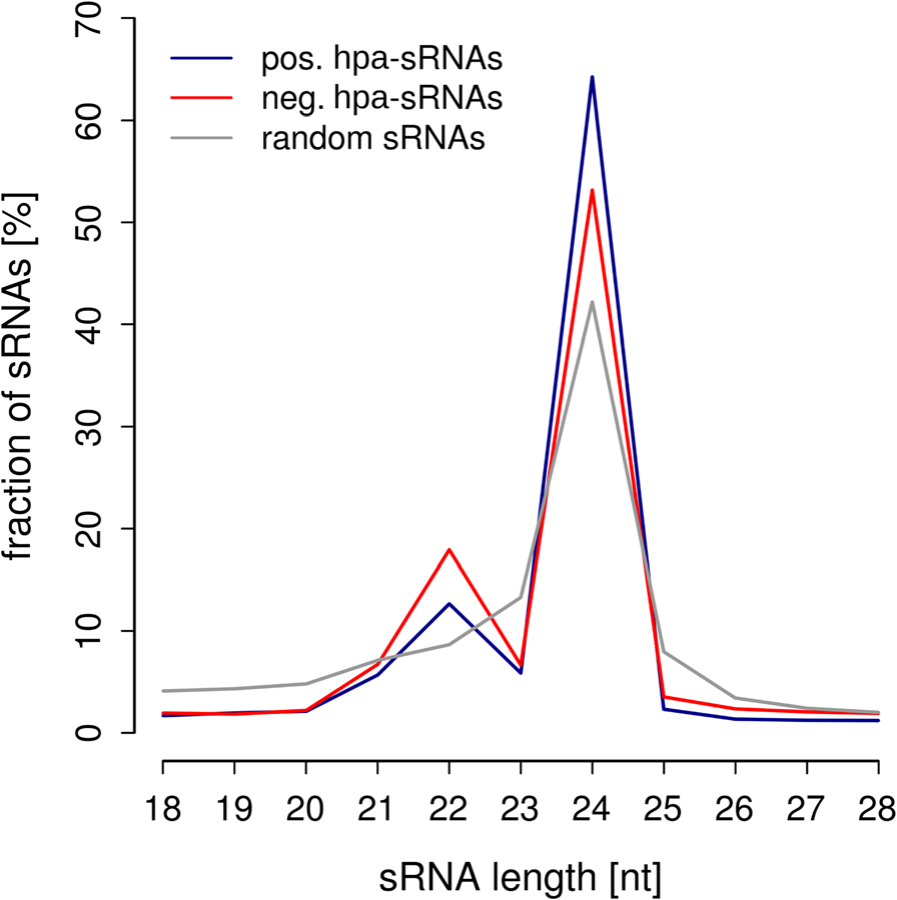
Enrichment of hpa-sRNAs for lengths of 22-nt and 24-nt. Size distribution of positively/negatively hpa-sRNAs and random sets of sRNAs. Enrichment analysis by bootstrapping (*p* < 0.001)

The high accuracy of sRNA-based predictions supports a functional relationship of the associated sRNAs with hybrid performance. First, we analyzed the size distribution of positively and negatively hpa-sRNA in relation to the whole set of sRNA sequences. Both classes of hpa-sRNAs are enriched for sRNAs with length of 22-nt and 24-nt (*p* < 0.05, bootstrap analysis, Fig. 5), which represent accepted functional size classes in maize [33]. Next, we tested positively and negatively hpa-sRNAs of canonical size classes (21-, 22-, and 24-nt) for homology to rRNAs, tRNAs, or miRNAs and found minor fractions between 0.07% and 10.18% of ha-sRNA overlapping with the first two sequence classes; no known microRNA was among hpa-sRNAs (Additional file 4: Table S3).

**Fig. 6:**
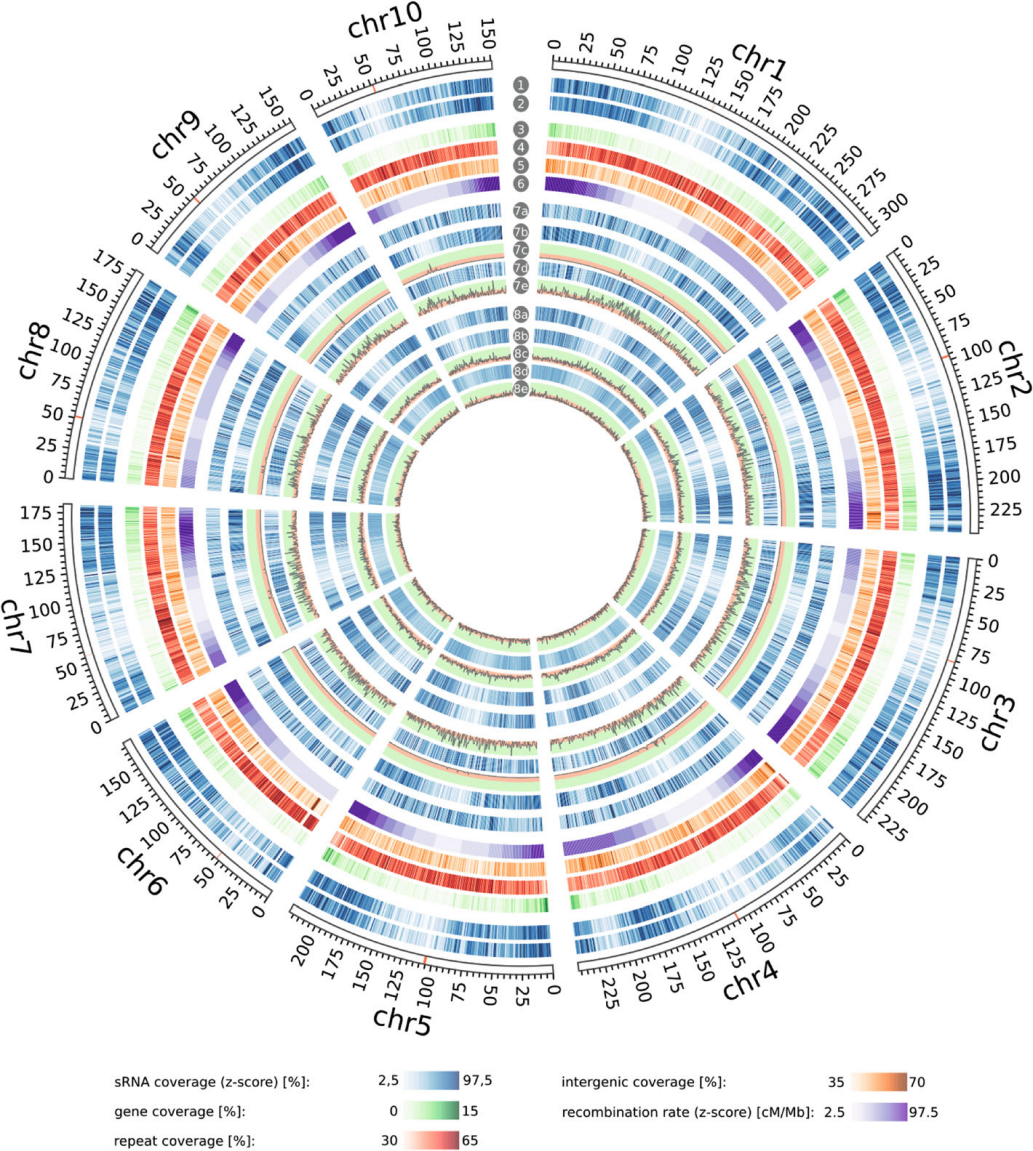
Genome-wide distribution and enrichment of sRNAs. Genomic coverage of hpa-sRNAs (1), all sRNAs (2), genes (3), repeats (4), intergenic regions (5) and recombination rates (6) throughout the B73 reference genome. Distribution of 22-nt sRNAs (7a), positively 22-nt hpa-sRNAs (7b), negatively 22-nt hpa-sRNAs (7d), 24-nt sRNAs (8a), positively 24-nt hpa-sRNAs (8b), negatively 24-nt hpa-sRNAs (8d) on the B73 reference genome. -log_10_ plot of enrichment probabilities of positively 22-nt hpa-sRNAs (7c), negatively 22-nt hpa-sRNAs (7e), positively 24-nt hpa-sRNAs (8c) and negatively 24-nt hpa-sRNAs (8e). Peaks in green background zone show significant enrichment (*p* < 0.05). All distributions are shown in 1 Mb resolution**.** Centromeres according to Jiao et al. [31] are indicated red in the rulers. Whole-genome visualization was created with Circos [43]. Annotations in (2) to (4) are according to genome assembly AGPv4.36

The genome-wide distribution of hpa-sRNAs overall resembles the distribution of all sRNA of the inbred line population. In contrast to all 24-nt hpa-sRNAs and 22-nt positively hpa-sRNAs, we identified the 22-nt negatively hpa-sRNAs as enriched in pericentromeric regions (p < 0.05, bootstrap analysis, Fig. 6). Their actual distribution is inversely correlated with the recombination rate on 8 out of the 10 maize chromosomes, ranging from − 0.37 for chromosome 5 to − 0.92 for chromosome 3 (Table 5).

**Table 5:** Correlation coefficients of genomic hpa-sRNA distributions with the recombination rate separated by different classes of hpa-sRNAs

	positively 22-nt	negatively 22-nt	positively 24-nt	negatively 24-nt
chr1	0,960	−0,770	0,957	0,964
chr2	0,961	−0,840	0,964	0,958
chr3	0,914	−0,915	0,918	0,859
chr4	0,952	−0,903	0,934	0,730
chr5	0,930	−0,365	0,930	0,902
chr6	0,972	0,134	0,932	0,951
chr7	0,908	−0,883	0,956	0,954
chr8	0,614	−0,607	0,491	0,656
chr9	0,822	-0,904	0,808	0,808
chr10	0,880	-0,032	0,842	-0,424

**Fig. 7:**
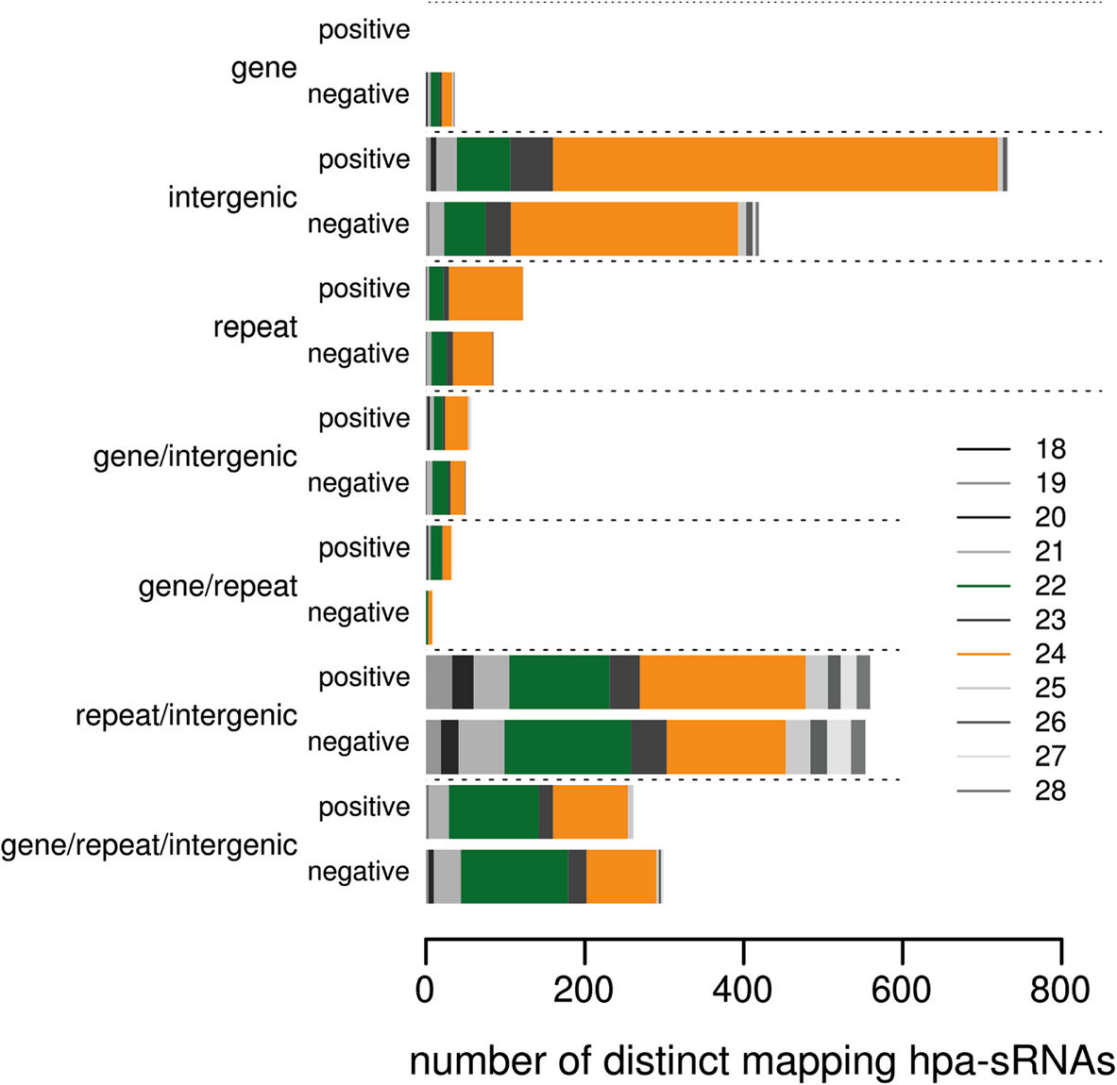
Relation of hpa-sRNAs to genomic features. Size distribution of hpa-sRNAs mapping to single or multiple annotated features of the maize genome; 22-nt hpa-sRNAs map primarily to multiple annotations (repeat/intergenic, gene/repeat/intergenic), while 24-nt hpa-sRNAs map primarily to single annotations (intergenic or repeat)

Finally, we explored the relationship of hpa-sRNAs to the annotated maize genome, subdivided into generally annotated features: 1) transcribed, protein coding sequences (gene); 2) TE or repeats (repeats); and 3) sequences without one of the previous annotations (intergenic). Whereas the majority of the 24-nt sRNAs map solely to intergenic regions of the genome, the 22-nt sRNAs map predominantly to multiple annotations (Fig. 7).

## Discussion

### Correlation of genomic and mRNA/sRNA expression differences with hybrid performance

We tested the correlation of genomic (SNP) and expression (mRNA, sRNA) differences between inbred lines with HP for GY in their hybrid offspring. It has already been shown that the genetic distance per se is not a sufficient predictor to determine HP or the extent of heterosis of an inbred line combination [2]. This finding holds true for the correlation of genomic differences in terms of SNPs between inbred lines with HP for GY (Fig. 1a), which resulted in a significant but moderate correlation. The correlation of mRNA differences with HP for GY resulted in a weak positive correlation and did not lead to improved results compared to genomic differences based on SNPs. Although the correlation of sRNA differences with HP for GY resulted in a slightly better correlation than for the SNP-based genetic distance, the notable difference is the inversion of the correlation. In contrast to SNP and mRNA expression differences, where increased differences coincided with higher HP for GY, the opposite was found for sRNAs expression differences. This overall negative correlation in the investigated population suggests, that less sRNA expression differences between the inbred parents might result in higher HP for GY. The inversed correlation suggests sRNAs to integrate different information, related to HP for GY, than provided by the genomic code (SNP) or the gene expression information (mRNAs). sRNAs have been shown to have functional roles in post-transcriptional gene regulation [34–36] as well as on the transcriptional level by modulating the epigenetic landscape [37, 38] after the hybridization of two distinct parental genomes. Additionally to the direct involvement of sRNAs in regulatory processes, they are themselves subjected to the transcriptional activity of the loci they are generated from and thus capture epigenetic and transcriptional information on genome-wide scale [32]. Hence, we assume that sRNAs are suitable markers to capture relations of parental differences with HP for GY by integrating information on genome-wide regulatory processes on top of genomic information represented by SNPs, as well as processes downstream of mRNA transcription represented by mRNA data.

### Association of differential data types with hybrid performance

To identify SNP, mRNAs or sRNAs with strong relation of parental differences and HP for GY, we employed an association approach based on the method described in Frisch et al. [5] with a modification to consider not only for positively associated markers, but as well for those who have a negative association with the trait of interest. The introduction of negative association was suggested by the negative correlation of parental sRNA expression differences with HP for GY (Fig. 1c). For all three data types negatively associated features were found. Whereas the sets of associated SNPs and sRNAs contain substantial fractions of negatively associated markers, mRNAs are predominantly positively associated (82.2%) with HP for GY. Contradictory to the correlation of sRNA expression differences with HP for GY, the negatively associated sRNAs with HP for GY represent only a minor fraction (38.1%). This may have two probable reasons, which are not mutually exclusive: The quantitative effect of negatively associated sRNAs on the phenotype might be stronger than for positively associated sRNAs, or negatively associated sRNAs may exhibit more extreme expression differences than positively associated sRNAs, thus dominating the correlation. It should be noted that in contrast to the correlation analyses of sRNA expression differences, the associations are not based on the actual quantitative differences but on about the frequency of differential expression. This property avoids the negligence of low expressed or overestimation of highly expressed sRNAs or mRNAs, since expression levels do not reflect protein levels or functional importance [39, 40].

The large numbers of associated individual markers of all three data types likely reflect HP for GY being a highly complex trait, which is most likely affected by many genomic loci each with small contributions to the phenotype. Considering as many components as possible that have an effect on HP for GY should thereby increase the prediction of this trait. In terms of breeding we assume that actively selecting against constraining elements, which are likely represented by negatively associated SNPs, mRNAs or sRNAs, might result in higher HP for GY.

### Correlation of marker-based distances with hybrid performance

The correlation of marker-based distances for associated markers with HP for GY resulted in strong and very strong correlations ranging in absolute values from 0.79 to 0.86. In general, we observed stronger correlations for mRNA and sRNA than for SNP based distances. We assume these higher correlations are caused by more information being integrated in mRNA and sRNA expression patterns in contrast to the pure genomic information from SNPs. mRNAs contain SNP information which is located in coding regions and might have effects on the protein function, but in contrast to SNPs provide additional information about transcriptional differences between the inbred lines. sRNAs are genome-wide regulators of the epigenetic landscape and themselves subject to the transcriptional activity regulated by the epigenetic state of their region of origin [32]. It is evident that only the combination of differing inbred lines can generate a better performing hybrid than its parents, by exploiting what is known as the heterosis effect [41]. Since both mRNAs and sRNAs harbor information of additional levels on differences between inbred parents, we expect them to have more explanatory and predictive power.

In a previous study, correlations of transcriptome data with HP for GY were performed based on the same mRNA dataset as in this study [5]. Our correlations with binary distance based on positively associated mRNAs (D_b,pos_) largely resemble the correlations with transcriptome-based binary distance in of Frisch et al. [5], but in contrast to the previous study our FDR adjusted *p*-value threshold was set to *p* < =0.05 instead of *p* < =0.01. This relaxation of the conditions for marker selection by binomial exact tests resulted in a slightly decreased correlation coefficient (0.82 instead of 0.86). However, we applied the threshold of *p* < =0.05 to allow for comparison of SNP, mRNA and sRNA data in the present study. Within our analyses we demonstrated that integrating negatively associated mRNAs increase the correlation coefficients. Positively associated mRNAs alone (D_b,pos_) resulted in r of 0.82, the combined binary distance for both negatively and positively associated mRNAs (D_b,com_) in r of 0.84, and the MLR in r of 0.86. With increased correlation coefficients higher predictive power is indicated.

### Hybrid prediction

Correlations of the observed with predicted HP for GY by 100 cross-validation runs, resulted in the expected behavior for all three data types (SNP, mRNA, sRNA) with stronger prediction accuracies for type-2 predictions than type-1 predictions and the least performance for type-0 predictions, for which test-cross data for both parents were lacking. The results show for all three data types that having one of the parents evaluated in test-crosses (type-1) results in considerably higher prediction accuracy with lower variability. The gained prediction accuracy by having both parents evaluated in test-crosses (type-2) is not as pronounced as it is between type-0 and type-1 prediction (Additional file 2: Figure S1A-C).

We compared our prediction approaches with those from a previous study that used the same mRNA dataset, but considered positively associated mRNAs only [5]. The statistics of our prediction using the binary distance of positively associated mRNAs (D_b,pos_) are identical to the statistics of the approach using the transcriptome-based binary distance of Frisch et al. [5] and both resulted in comparable prediction accuracies. Inclusion of information on negatively associated mRNAs improved the prediction accuracy for type-2 and type-1 but not type-0 predictions. For type-0 predictions the positively associated mRNAs (D_b,pos_) resulted in highest prediction accuracies (Additional file 2: Figure S1B). Importantly, both predictions that include the negatively associated mRNAs, integrated in combined binary distance (D_b,com_) or the MLR-based prediction resulted in considerably less variation of the prediction accuracy (Additional file 2: Figure S1B). These results reveal, that the introduction of the negatively associated mRNAs considerably increases the type-2 prediction accuracy for HP for GY by mRNA expression profiles. For type-1 prediction, where one of the parental lines has been evaluated in test-crosses, the combined binary distance (D_b,com_) outperformed the binary distance for positively associated mRNAs (D_b,pos_). This again highlights that information on negative contributions to HP for GY are important for the prediction accuracy. That type-0 predictions based solely on the binary distance of positively associated mRNAs (D_b,pos_) could not be improved by adding additional information about negatively associated mRNAs (D_b,com_, MLR-based prediction) suggests that information from related crosses, with shared parental lines, are important to select most informative individual markers.

Using SNP and sRNA as markers the MLR-based predictions resulted in highest prediction accuracies for type-2 predictions. The combined binary distance (D_b,com_) based predictions outperformed all other approaches for type-1 predictions. Clearly, like for predictions using mRNA expression profiles, the integration of negatively associated markers facilitates a more precise selection of the best inbred line combinations. This holds true, as long as test-crosses for at least one of the parents have been supplied in the estimation set of the prediction model. In the case the estimation set is composed solely by unevaluated lines (type-0), the MLR prediction approach showed a poor performance. Thus nor the information added in the two predictor variables m_f_ and m_d_ neither the separate binary distances for positively and negatively associated markers (D_b,pos_, D_b,neg_) provide a gain of precision anymore. We assume that a lack of germplasm in the estimation set with genomic relation to the inbred line combinations that are supposed to be predicted hampers the identification of the predictors needed for accurate prediction. Although the prediction accuracies for type-0 predictions were low and highly variable for the negatively associated markers (D_b,neg_), the predictions using the combined binary distance (D_b,com_) performed similar or better than predictions based on positively associated markers (D_b,pos_) alone. The prediction approach using the combined binary distance (D_b,com_) as predictor was thus shown to be less susceptible to the composition of the estimation set.

Overall the prediction accuracies of the cross-validation runs revealed that predictions based on sRNA expression performed better than mRNA expression and SNP profiles for both the combined binary distance (D_b,com_) and the MLR-based predictions (Fig. 3a-c). Although the combined marker-based predictions resulted in a slightly lower performance in comparison to the MLR-based prediction in type-2 predictions, overall, including type-1 and type-0 prediction, the combined marker-based distance resulted in a better and more stable performance. Thus a MLR-based prediction might be beneficial only for the selection of breeding lines with a high number of tested lines or very closely related breeding material.

The prediction models rely on differences between parents of different heterotic groups. Our strategy to measure RNA expression differences across the population involved pooling of individually grown plants of each inbred line to reveal genotypic effects and to average environmental effects and did not include biological replicates. Thus we used simple thresholds to call differentially expressed sRNAs for the identification of trait-associates ones by binomial testing. By DESeq2 with related lines set as replicates we confirmed sRNA expression differences between the heterotic groups. In addition, the high congruence in the relation of positively and negatively hpa-sRNAs called by the various methods in two sets of inbred lines supported the validity of threshold based differential expression of sRNAs. Further support for the validity of the threshold based sRNA expression analysis may be derived from the expression analysis of known miRNAs. Consistently, no known miRNAs where among the hpa-sRNAs and the number of miRNAs identified by DESeq2 was very low. Given that most miRNAs are developmentally or environmentally regulated [33] the low number of differentially expressed miRNAs is in agreement with the identical developmental stage and growth conditions of the sampled seedlings.

### Characteristics of sRNAs associated with hybrid performance for grain yield in maize

Hpa-sRNAs are enriched for sRNAs with length of 22-nt and 24-nt and thus mainly represent size classes with implicated function in maize [33], which are likely to be generated by different pathways of biogenesis [42]. Our tests for homology of hpa-sRNAs to highly abundant tRNAs and rRNAs do not provide evidence to support direct relations of parental expression variations with hybrid performance for sRNA sequences derived from these RNA classes.

The preferential mapping of 24-nt hpa-sRNAs to just single features indicates that they may have restricted spatial activity, primarily acting on specific loci at their site of origin, whereas the high proportion of 22-nt hpa-sRNAs mapping to multiple features point to their potential of *trans*-regulatory action on functional genes distant from the site of origin. Importantly both hpa-sRNA classes have the potential of trans-allelic action in the hybrid genome.

## Conclusions

Hybrid prediction has the potential to both improve hybrid breeding by speeding up and enhance the selection of most promising inbred line combinations and reducing the requirement of expensive field trials. In this study we developed a sRNA-based prediction approach of hybrid traits. For this purpose we advanced an association approach to identify also negatively - in addition to positively - trait-associated markers. We propose two prediction approaches, which integrate the information about positively and negatively associated markers and evaluated the prediction accuracy using sRNA as markers in comparison with SNP and mRNA based predictions. We showed that sRNA-based predictions are highly accurate when test-crosses are available for some of the tested parents and that the integration of negatively associated markers improve the prediction accuracies for all three analyzed data types (SNP, mRNA, sRNA). The genomic characteristics of the hpa-sRNAs we identified indicate a functional contribution of these sRNAs to the formation of hybrid performance.

## Additional files

Additional file 1: **Table S1:** Hybrid performance for grain yield of 7x14 Flint/Dent crosses measured in Mg ha^-1^ adjusted to 155 g kg^-1^ grain moisture. (XLS 16 kb)

Additional file 2: **Table S2:** Summary of sRNA sequencing data. (XLS 20 kb)

Additional file 3: **Figure S1:** Comparison of prediction accuracies of different prediction types. (A) SNP, (B) mRNA, (C) sRNA based predictions. (TIF 9480 kb)

Additional file 4: **Table S3:** Number and percentage of hpa-sRNAs with homology to known miRNA, tRNA and rRNA. (XLS 27 kb)

## Abbreviations

DE: Differentially expressed
epiRIL: Epigenetic recombinant inbred lines
FDR: False Discovery rate
GY: Grain yield
HP: Hybrid performance
MLR: Multivariate linear regression
mRNA: Messenger RNA
SNP: Single Nucleotide polymorphism
sRNA: Small RNA
