# Supplementary figures and images for: Small RNA-based prediction of hybrid performance in maize
Source: BMC Genomics. 2018 May 21;19:371. doi: 10.1186/s12864-018-4708-8 (PMC5963143; doi:10.1186/s12864-018-4708-8)

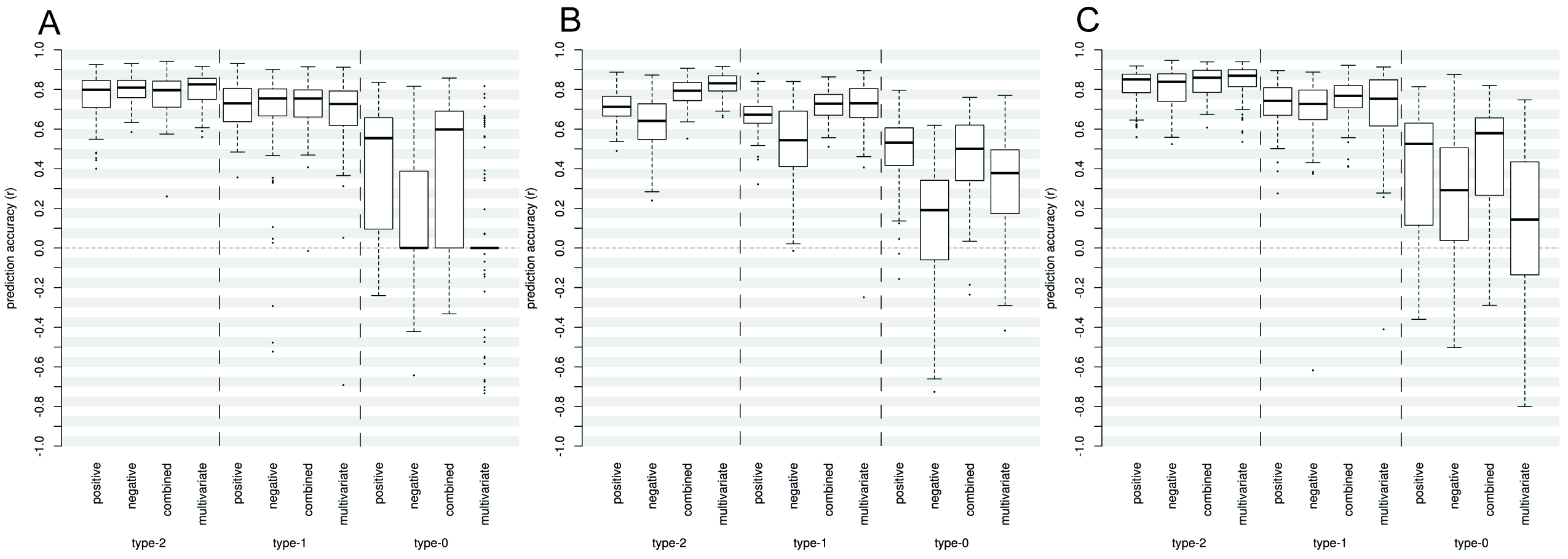

Supplement: Supplementary file 3 — Figure S1: Comparison of prediction accuracies of different prediction types. (A) SNP, (B) mRNA, (C) sRNA based predictions. (TIF 9480 kb) [file 12864_2018_4708_MOESM3_ESM.tif]
